# Preclinical models of treatment-resistant depression: challenges and perspectives

**DOI:** 10.1007/s43440-023-00542-9

**Published:** 2023-10-26

**Authors:** Magdalena Kolasa, Agata Faron-Górecka

**Affiliations:** Department of Pharmacology, Maj Institute of Pharmacology, Polish Academy of Sciences, Smętna 12, 31-343 Kraków, Poland

**Keywords:** Treatment-resistant depression, Animal models, Chronic mild stress, Wistar Kyoto rats, Ketamine, Psilocybin, DBS

## Abstract

Treatment-resistant depression (TRD) is a subgroup of major depressive disorder in which the use of classical antidepressant treatments fails to achieve satisfactory treatment results. Although there are various definitions and grading models for TRD, common criteria for assessing TRD have still not been established. However, a common feature of any TRD model is the lack of response to at least two attempts at antidepressant pharmacotherapy. The causes of TRD are not known; nevertheless, it is estimated that even 60% of TRD patients are so-called pseudo-TRD patients, in which multiple biological factors, e.g., gender, age, and hormonal disturbances are concomitant with depression and involved in antidepressant drug resistance. Whereas the phenomenon of TRD is a complex disorder difficult to diagnose and successfully treat, the search for new treatment strategies is a significant challenge of modern pharmacology. It seems that despite the complexity of the TRD phenomenon, some useful animal models of TRD meet the construct, the face, and the predictive validity criteria. Based on the literature and our own experiences, we will discuss the utility of animals exposed to the stress paradigm (chronic mild stress, CMS), and the Wistar Kyoto rat strain representing an endogenous model of TRD. In this review, we will focus on reviewing research on existing and novel therapies for TRD, including ketamine, deep brain stimulation (DBS), and psychedelic drugs in the context of preclinical studies in representative animal models of TRD.

## Introduction

Major depressive disorder (MDD) is a psychiatric condition characterized by a persistent low mood, cognitive impairment, and loss of interest or pleasure lasting for at least 2 weeks. According to WHO estimates, depression is projected to become the leading cause of disease burden worldwide by 2030. Mental disorders account for approximately 25.3% and 33.5% of all years lived with disability in low- and middle-income countries, respectively [1]. Within this spectrum, a subset of patients are classified as having treatment-resistant depression (TRD). TRD is defined as the lack of sufficient remission of depressive symptoms after at least two trials of antidepressant treatment with appropriate doses and duration. However, the prevalence of TRD is challenging to determine due to the absence of a universally accepted scale to assess treatment-resistant depression. Available questionnaires, such as Beck Depression Inventory, Hamilton Depression Rating Scale, and Montgomery–Asberg Depression Rating Scale [2–4], assess the severity of depressive symptoms but are not specifically tailored to define treatment resistance. Therefore, estimates of TRD prevalence vary significantly, ranging from 12 to 55% [5, 6], primarily due to the lack of a widely accepted TRD definition and consistent criteria used for its determination. The term pseudo-resistance [7] was used to refer to the lack of response to inadequate treatment in terms of duration or dose of antidepressants, but it seems that more factors can cause the so-called pseudo-resistance depression [5]. Despite numerous definitions and classification models of TRD in the literature, a consensus on standardized criteria for assessing and defining TRD remains elusive. Therefore, the aim of this article is to provide a comprehensive analysis of the challenges associated with TRD diagnosis, underscore the multifactorial nature of TRD, evaluate the potential of animal models in TRD research, and review current and innovative therapeutic strategies for this condition. The goal of the article is to contribute to better understanding and treatment of TRD, which represents a significant challenge in contemporary pharmacology and psychiatry.

## Criteria diagnosis of TRD

Clinical diagnosis of TRD often relies on the observation and the analysis of a patient's treatment history. Psychiatrists thoroughly evaluate a patient's response to various antidepressants and psychotherapies, using relevant questionnaires to classify treatment-resistant depression based on severity and resistance to antidepressant treatment. Several available methods, such as Thase and Rush Staging Methods [8], European Staging Method (ESM) [5], Massachusetts General Hospital Staging Method (MGH Staging Method) [9], as well as guidelines developed within Sequenced Treatment Alternatives to Relieve Depression (STARD) [10], and Canadian Network for Mood and Anxiety Treatments (CANMAT) Guidelines [11], aim to classify treatment resistance in depression.

Thase and Rush Staging Methods, ESM, and MGH Staging Method are distinct approaches to classifying treatment resistance in treatment-resistant depression. While all these methods aim to determine the level of resistance to antidepressant treatment, they differ in their approach and assessment criteria. Thase and Rush Staging Methods focus on evaluating a patient's response to two or more trials of antidepressant treatment, classifying patients based on the degree of resistance in two or three stages (e.g., mild and moderate resistance). ESM emphasizes complete remission of depressive symptoms as the primary criterion for treatment success, requiring patients to undergo at least four different trials of antidepressant treatment with appropriate dose and duration and classifying them into three stages of resistance (mild, moderate, and severe). The MGH Staging Method assesses various levels of resistance to treatment, including mild, moderate, and severe resistance, and requires patients to undergo at least two trials of antidepressant treatment before being classified as treatment-resistant (Table 1).

**Table 1:** Comparison of treatment-resistant depression level classification systems based on [5, 8]

	Classification of treatment resistance level according to Thase and Rush method
Stage 0	Previous treatment attempts were deemed insufficient
Stage I	Failure of at least 1 appropriate medication from the main class of antidepressant drugs
Stage II	Failure of at least 2 attempts with two distinctly different classes of antidepressant drugs
Stage III	Stage II plus failure of tricyclic antidepressant therapy
Stage IV	Stage III plus the failure of monoamine oxidase inhibitors (MAOIs) therapy
Stage V	Stage IV plus bilateral electroconvulsive therapy

	European classification of treatment resistance level	
Lack of response to treatment	Tricyclic antidepressant Selective serotonin reuptake inhibitor Dual serotonin and norepinephrine reuptake inhibitors (e.g., venlafaxine) Electroconvulsive therapy Other antidepressant drugs Lack of response to an adequate trial with an antidepressant drug	Duration of treatment trial: 6–8 weeks
Treatment-resistant depression (TRD)	Resistance to 2 or more appropriate treatment trials with antidepressant drugs	Duration of trial periods: TRD 1: 12–16 weeks TRD 2: 18–24 weeks TRD 3: 24–32 weeks TRD 4: 30–40 weeks TRD 5: 36 weeks–1 year
Chronic TRD	Resistance to multiple trials of antidepressant drug treatment, including augmentation strategy	Duration of trial period: at least 12 months

In conclusion, these three methods differ in their approach to assessing treatment resistance in treatment-resistant depression, employing distinct criteria and classifications. Each method aims to provide a better assessment and understanding of a patient’s resistance to antidepressant treatment, aiding in the customization of further therapeutic strategies. Nevertheless, the varying criteria for TRD assessment contribute to the disparate prevalence estimates of treatment-resistant depression.

## Factors contributing to treatment resistance in depressed patients

What are the causes of TRD? Unfortunately, there is no clear answer to this question. TRD can be influenced by several other factors that are complex and individual for each patient. Among the main factors that can contribute to the lack of response in depressed patients to standard antidepressant therapies are:

### Individual biological diversity

Individuals with depression have varying biological, genetic, and neurochemical characteristics that can influence their response to antidepressant medications. Despite the lack of available biological and genetic markers for TRD, it appears that refining genome-wide association study (GWAS) investigations holds the potential to identify clinically relevant genetic biomarkers. Studies employing GWAS genotyping methods targeting polymorphisms within genes known to be associated with mechanisms of antidepressant drug action (pharmacodynamics) or metabolism (pharmacokinetics) have revealed several potential candidate genes implicated in TRD [12].

Genetic investigations within the realm of Treatment-Resistant Depression (TRD) are centered around the identification of genes linked to glutamatergic and monoaminergic neurotransmission, alongside the modulation of synaptic plasticity. Notably, pivotal genetic determinants include those associated with the N-Methyl-D-Aspartate (NMDA) receptor, notably the genes GRIN2B (glutamate ionotropic receptor NMDA type subunit 2B) and GIRK4 **(**glutamate ionotropic receptor kainate type subunit 4). These genes play a crucial role in shaping the responsiveness to interventions targeting depression, such as ketamine administration and electroconvulsive therapy (ECT). Additionally, alterations in peripheral brain-derived neurotrophic factor (BDNF) expression have been observed in TRD patients, revealing a decrease compared to those who exhibit a responsive reaction to antidepressant treatments. This finding aligns with the theoretical framework proposing a deficit of BDNF within the broader context of MDD and notably in the specific context of TRD [13]. BDNF also plays a significant role in TRD pathogenesis, with a specific focus on the rs6265 polymorphism. Preclinical research [14] and a small clinical pilot study primarily conducted in European populations [15] suggest that the presence of the Met allele weakens the antidepressant response to ketamine in TRD. However, a subsequent study conducted on a Chinese population did not confirm this hypothesis and demonstrated that TRD patients exhibited dose-dependent ketamine efficacy irrespective of the rs6265 genotype [12, 16].

PPP3CC (protein phosphatase 3 catalytic subunit gamma) may have a role in the activation of a neuron-enriched phosphatase that regulates synaptic plasticity [17]. It has been suggested as a candidate for TRD risk [18], possibly through an interaction with BDNF and serotonin receptor type 2S (5-HT2AR) polymorphisms as reported above [19]. Monoaminergic genes, such as SLC6A4 (solute carrier family 6 member 4), SLC6A2 (solute carrier family 6 member 2), 5-HT2AR, 5-HT1AR (serotonin receptor type 1), and COMT (catechol-*O*-methyltransferase), have also been investigated as potential TRD risk factors, although results have often been conflicting or negative. Additionally, the 5-HTTLPR (serotonin-transporter-linked polymorphic region) polymorphism in the SLC6A4 gene has a controversial association with TRD, and its impact on treatment response remains uncertain [12]. It has also been demonstrated that genes associated with immune response, such as the Leukotriene B4 Receptor (LTB4R) and Complement C1q Receptor (CR1L), may be linked to the response to antidepressant treatment. Additionally, genes involved in synaptic plasticity regulation, such as Synaptic Vesicle Glycoprotein 2A (SV2A) and Complement Component 3 (C3), have also been observed to potentially influence treatment response in depression. However, due to study limitations, further replication studies are necessary to confirm these genetic associations [20].

Regarding the pharmacokinetics of antidepressants, genes of the cytochrome P450 (CYP450) family and the P-glycoprotein (P-gp) encoded by the ATP-binding cassette subfamily B member 1 (ABCB1 gene) are relevant for drug metabolism and transport. Functional variants in the CYP2D6 (cytochrome P450 2D6) and CYP2C19 (cytochrome P450 2C19) genes have the potential to influence TRD risk, while the P-gp protein may affect treatment response by regulating the transport of antidepressants across the blood–brain barrier [21].

Recently, the hypothesis postulating an association between infrequent, potentially functional genetic variants and TRD has been subjected to investigation [22]. This study employed whole exome sequencing data obtained from a cohort of 149 TRD cases, and subsequent analysis aimed to discern an excessive accumulation of infrequent genetic variants. At the gene level, an ensemble of five genes—namely, ZNF248 (zinc finger protein 248), PRKRA (protein activator of interferon-induced protein kinase), PYHIN1 (pyrin and HIN domain family member 1), SLC7A8 (solute carrier family 7 member 8), and STK19 (serine/threonine kinase 19)—exhibited a statistically robust surplus of variants within TRD cases. The scrutiny of 41 pre-selected gene sets provided indications of an augmented prevalence of infrequent, functional variants within genes implicated in the response to lithium. Remarkably, among the genes previously identified in TRD investigations, ZDHHC3 (zinc finger DHHC-type palmitoyltransferase 3) also demonstrated significance within this dataset, accounting for multiple testing corrections. ZNF248 and STK19 are implicated in the regulation of transcription processes, while PYHIN1 and PRKRA play roles in immune response modulation. SLC7A8 is associated with thyroid hormone transporter activity, and ZDHHC3 governs the synaptic clustering of GABA and glutamate receptors. These findings lend support to the assertion that infrequent, functional alleles hold relevance within the context of TRD, thereby providing direction toward promising avenues for forthcoming research endeavors [22].

In summary, genetic research provides insights into potential genes and mechanisms associated with TRD. However, many of these conclusions require further investigation and replication in larger samples to achieve more definitive and robust results.

### Co-existing medical conditions

The presence of chronic illnesses significantly elevates the risk of developing depression, with depressive disorders being approximately twice as prevalent among individuals with conditions, such as diabetes, coronary artery disease, HIV infection, and stroke, compared to those without chronic illnesses [23]. Conversely, depression has been found to heighten the risk of developing various chronic medical conditions. For instance, depression has been associated with a twofold increase in the risk of type 2 diabetes and a 64% higher risk of coronary artery disease [24]. Hence, the presence of concurrent medical conditions can also affect the efficacy of antidepressant therapy. An important factor in this regard is the potential for misdiagnosis, which may result in inadequately tailored treatment due to the wide spectrum of depressive symptoms. Furthermore, pharmacodynamics and pharmacokinetics factors may also contribute to pseudo-resistance depression [5]. Several studies have linked TRD to lower plasma/serum levels of tricyclic antidepressants [5]. For instance, the concurrent use of metabolic inducers (such as drugs that can enhance the metabolism and elimination rate of co-administered agents) may lead to a relative reduction in antidepressant blood levels, resulting in an inadequate response. Conversely, some medications used to treat other medical conditions can elevate the levels of ADs, potentially resulting in bothersome side effects and patient discontinuation of treatment [25].

A study examining patients suffering from TRD compared to patients with depression who respond to treatment (MDD) has revealed significant associations between TRD and various physical illnesses [26]. TRD patients were found to be more susceptible to multiple health issues, such as chronic obstructive pulmonary disease, kidney disease, rheumatoid arthritis, or heart disease. Previous investigations focusing on Danish patients with TRD have revealed an augmented frequency of antecedent general medical conditions affiliated with the immune or neurological systems, musculoskeletal disorders, and migraines. In tandem, subsequent maladies exhibited an expanded range, encompassing cardiovascular, endocrine, and neurological disorders [27]. These findings suggest that TRD patients have a higher risk of coexisting physical conditions, underscoring the need for a comprehensive approach to their care that considers psychological and physical aspects.

### Underestimation of dosage or treatment duration

At times, patients do not receive the appropriate medication dosage, or the treatment duration is too short, which can affect the response to therapy. Furthermore, the pharmacotherapy of antidepressants is associated with a multitude of side effects, thereby frequently prompting patients to reduce the recommended dosages of antidepressant medications to mitigate adverse effects. Also typically, the determination of suitable treatment duration is based on industry-sponsored trials focused on establishing significant distinctions between medications and placebos. However, for individuals with TRD, the optimal duration of antidepressant therapy might surpass the standard 4- to 6-week span employed in these trials [5]. Consequently, prolonged trials extending beyond 10 weeks, particularly in refractory cases, have been proposed to potentially elicit therapeutic responses [28, 29]. Moreover, among elderly individuals who suffer from depression, a timeframe exceeding 12 weeks might be essential for substantial clinical amelioration [5, 29].

A comprehensive analysis of clinical trials about TRD revealed a notable absence of consensus in delineating treatment outcomes as successful or unsuccessful grounded in maximum dosage thresholds. A majority of investigators either omitted these thresholds or employed generic terminology like “adequate doses” or “acceptable therapeutic doses.” It is of significance to underscore those investigations furnishing specific data concerning tricyclic antidepressants (TCAs) exhibited substantial disparities in the stipulated minimal dosage requisites, thereby accentuating incongruities in the established standards for treatment adequacy. Analogous disparities were discerned in studies centering on selective serotonin reuptake inhibitors (SSRIs), wherein divergent dosage spectra emerged as prerequisites across distinct investigations, further underscoring the exigency for standardized protocols to ascertain treatment efficacy contingent upon dosage considerations [29].

### Psychosocial factors

Factors, such as stress, trauma, social support, and lifestyle, can influence the effectiveness of depression treatment. TRD patients were demonstrated to have some distinctive clinical features compared with non-TRD patients, such as higher symptom severity, more frequent suicidal risk, and comorbidity with anxiety [30]. TRD patients often face a higher incidence of significant life stressors, including events, such as immigration, family bereavement, interpersonal conflicts, job termination, financial strains, serious health problems, and life-threatening situations [31]. There is also evidence suggesting that TRD is associated with the “melancholic” subtype of depression, as a high prevalence of this subtype has been observed in ambulatory TRD patients [32]. The melancholic subtype historically differs from other types of depression by affective disturbances that are disproportionate or without cause, psychomotor retardation, and cognitive impairments [33].

Despite the complexity of this disorder and the many factors that can influence it, research into TRD is extremely important. It seems that animal models of TRD provide an opportunity to study not only the mechanisms underlying treatment-resistant depression but also new potential therapies.

## Animal models in research on treatment-resistant depression

Currently, there is a lack of unequivocal biomarkers that could predict the effectiveness of treatment for individual patients, leading to a trial-and-error approach in drug selection. Consequently, there is a need to develop new translational models to better comprehend the neurobiological mechanisms of depression and treatment efficacy. In reviewing the latest advancements, researchers are emphasizing the search for predictive biomarkers that could assist both in clinical practice and in clinical trials of novel compounds. The investigation carried out on patient cohorts should aim to provide profound insights into potential biomarkers associated with this disorder. Nevertheless, the intricate nature of TRD described above, coupled with the absence of standardized criteria, poses a distinctive challenge for researchers. Animal models offer a robust avenue for diagnosing and exploring novel biomarkers relevant to TRD. Studies involving animal populations that similarly manifest non-responsiveness to antidepressant interventions offer a more controlled research environment, circumventing potential confounders like concurrent medical conditions or supplementary pharmacotherapy, which could introduce bias to the TRD landscape.

A good animal model can be described from the perspective of its: (a) similarity (face validity—similarity of factors that induce the disease and human symptoms), (b) predictability (predictive validity—response and similarity of the response to standard clinical treatment), (c) mechanism (construct validity—similarity of physiological or psychological disease mechanisms). Unfortunately, animal models often only exhibit superficial similarity (face validity) reflecting processes different from those present in the clinical situation [34].

However, there are several well-established animal models of depression, including Learned Helplessness (LH), Early life stress model, Olfactory bulbectomy (OBX) model, Social defeat model, Chronic restraint stress model, Glucocorticoid/corticosterone model, or Genetic model of depression [35, 36] followed by behavioral tests related to anhedonia (e.g., sucrose preference) or behavioral despair [e.g., forced swim test (FST) and tail suspension test (TST)]. However, the latter often respond to acute antidepressant treatment and do not encompass many aspects necessary for an effective animal model of depression (Table 2).

In the realm of investigating TRD, notable attention is drawn to the Chronic Mild Stress (CMS) model. First introduced by Katz [37], this model exposes rats to a range of highly stressful stimuli over several weeks [38]. The ramifications of chronic stress are assessed using metrics like sucrose consumption, wherein rats subjected to prolonged stress exhibit diminished intake. Concurrently, these animals manifest reduced responsiveness to pleasurable stimuli, commonly referred to as anhedonia, a cardinal facet of depression. Additional depressive symptoms, including diminished social interactions, cognitive impairments, heightened anxiety, and alterations in sleep patterns, have been observed in stressed subjects. Throughout scientific literature, diverse iterations of the CMS model are denoted by distinct terms, such as chronic unpredictable stress (CUS), chronic unpredictable mild stress (CUMS or UCMS), or chronic variable stress (CVS). Despite nomenclatural variances, the methodologies themselves generally exhibit marked similarities and negligible deviations from one another [39].

The CMS model has been corroborated as a viable animal representation of depression, underscored by robust constructs, congruence with observable symptoms, and predictive correlations. Its responsiveness to diverse antidepressants and agents endowed with anti-anhedonic attributes further bolsters its relevance. Furthermore, within the realm of animals subjected to the CMS procedure, approximately 30% of the animals do not respond to antidepressant treatment [40, 41]. Consequently, it appears that the group of treatment-resistant animals corresponds to the population of patients non-responsive to antidepressant treatment and can serve as a highly effective model for TRD. Using this model, a negative correlation has been demonstrated between the behavioral response to pharmacotherapy in the CMS model and the basal level of prolactin (PRL). Given the described correlation between hyperprolactinemia, depression symptoms, and concurrent drug resistance, a hypothesis was formulated suggesting that the baseline PRL level might underlie therapy ineffectiveness [41].

Another noteworthy animal model of TRD is the Wistar Kyoto (WKY) rat strain. The origin of the WKY strain can be traced back to the year 1971 when its establishment aimed to provide a normotensive control counterpart for the spontaneously hypertensive rat (SHR) [42] investigations involving WKY and WIS cohorts have elucidated a distinct inclination of the WKY strain toward heightened sensitivity to stress [43], which has subsequently led to its recognition for susceptibility to stress-induced ulcers [44–47]. In comparison to other rat strains, WKY rats exhibit pronounced depression-like behavior characterized by heightened immobility in the FST, augmented anxiety-like behavior, and diminished activity in novel environments [48, 49]. Harnessing this strain across a spectrum of research endeavors centered on depression, WKY rats have emerged as a genetic archetype of endogenous TRD, exhibiting several notable congruencies with the human condition [50–54] (Table 2).

**Table 2:** Animal models in depression and treatment-resistant depression research and their characteristics [35, 36]

Animal model	Characteristics
Learned helplessness (LH)	Used to model depressive-like behavior; often responsive to acute antidepressant treatment but may not encompass all aspects of depression
Early life stress model	Involves exposure to stress early in life to induce depression-like symptoms in adulthood
Olfactory bulbectomy (OBX)	Surgical removal of olfactory bulbs to induce depressive behavior; limited in capturing all aspects of depression
Social defeat model	Involves chronic social stress leading to depressive-like behavior; may not represent all features of depression
Chronic restraint stress	Involves prolonged exposure to restraint stress to induce depressive-like symptoms; widely used
Glucocorticoid/corticosterone model	Uses stress hormones to induce depression-like behavior; relevant to stress-induced depression
Genetic model of depression	Based on specific genetic manipulations to create depressive-like behavior in animals
Chronic mild stress (CMS)	Exposes animals to various stressful stimuli over weeks, leading to depressive-like symptoms; responsive to antidepressants, although 30% of rats do not respond to ADs treatment, they therefore constitute the TRD group
Wistar Kyoto rat (WKY)	A genetic model with heightened sensitivity to stress; TRD model in compare to WIS Han rats; exhibits depression-like behavior and unresponsive to ADs, except ketamine and DBS

## What are the potential therapies for TRD?

Until recently, among the available therapies approved by the U.S. Food and Drug Administration (FDA) were: a combination therapy of two drugs—fluoxetine and antipsychotic medication olanzapine (Symbyax), electroconvulsive therapy (ECT), repeated transcranial magnetic stimulation (rTMS), and Vagus Nerve Stimulation (VNS) [55, 56]. Recently, esketamine (Spravato, Janssen Pharmaceuticals, Raritan, NJ) has been approved by FDA and the European Medicines Agency (EMA) as the only pharmacological agent with glutamatergic neuromodulatory properties aimed at enhancing the effects of selective serotonin reuptake inhibitors or serotonin and norepinephrine reuptake inhibitors [57, 58]. Despite the approval of this medication for TRD therapy, the conducted clinical studies have sparked controversy, particularly in the context of the employed definitions of TRD [59]. Regrettably, the utilization of esketamine engenders a spectrum of diverse adverse effects encompassing the potential for addictive proclivity, profound psychotomimetic manifestations, lower urinary tract disturbances, and pronounced cardiovascular perturbations. These untoward consequences impart substantial constraints upon its application, confining its prescription exclusively to patients under hospital supervision and necessitating concomitant administration with an oral antidepressant [60]. In the context of high rates of partial effectiveness or lack of response to currently available antidepressant drugs, multiple mechanisms of action of new pharmacological substances are being investigated, going beyond the stimulation of monoaminergic neurotransmitters. However, despite many compounds being studied in phases II and III of clinical trials, it is difficult to predict which of them will reach the market in the coming decades [61]. So far, only esketamine and brexanolone, the latter being a positive allosteric modulator of the γ-aminobutyric acid (GABA)-A receptor, are antidepressants with non-monoaminergic effects, approved by the FDA for supervised use in patients with TRD and postpartum depression, respectively [62].

Recent studies have been concentrating on the antidepressant effects of psilocybin in the context of TRD therapy. Psilocybin, a tryptamine alkaloid present in various species of psilocybe mushrooms, has been indicated for its potential antidepressant effectiveness through initial investigations involving patients with life-threatening cancer [63–65]. Previous pilot studies focusing on major depressive disorder, which encompassed comparisons between psilocybin and escitalopram [66] along with an exploration of its utility in treatment-resistant depression [67], have provided insights into the therapeutic prospects of this compound.

In February 2023, there were 69 ongoing clinical trials worldwide utilizing psilocybin, with 15 focusing on TRD. One clinical trial focusing on TRD patients was conducted at 22 sites in 10 countries in Europe (the Czech Republic, Denmark, Germany, Ireland, the Netherlands, Portugal, Spain, and the United Kingdom) and North America (Canada and the United States) from March 1, 2019, through September 27, 2021. Data from this study, when psilocybin monotherapy was administered over up to 12 weeks for patients grappling with TRD, revealed that the 25 mg dose demonstrated a significantly superior reduction compared to the 1 mg dose. However, no noteworthy difference emerged between the 10 mg dose and the 1 mg dose. Alongside common side effects, such as headaches, nausea, dizziness, and fatigue, some participants experienced suicidal ideation or self-injurious behavior, and this was more pronounced in the 25 mg and 10 mg groups compared to the 1 mg group. Notably, considering participants exhibiting a deterioration in suicidal disposition, the trial underscores the importance of clinical vigilance in potential future trials involving psilocybin for depression [68, 69]. Based on clinical trials data, on July 1, 2023, psilocybin was legalized by the Therapeutic Goods Administration (TGA) in Australia for the treatment of TRD [70].

Evidence from rodent studies demonstrates that not only psilocybin [71] but also other classical psychedelic substances, such as Lysergic acid diethylamide (LSD) [71, 72], psilocin, and *N*,*N*-dimethyltryptamine (DMT) [73], elicit enduring behavioral outcomes akin to those achieved with conventional antidepressant treatments, especially concerning coping strategies and cognitive functions. Furthermore, insights from animal research suggest that psychedelic compounds might enhance associative learning [72], a cognitive aspect often compromised by neuropsychiatric disorders. However, the literature addressing the consequences of psychedelic substances on rodent behavior in the context of psychiatric and cognitive functions is limited, and the outcomes of diverse investigations may appear harmonious or conflicting without true comparability [73]. A single administration of psilocybin (1 mg/kg) has been demonstrated to significantly modulate long-term behavioral parameters in male WKY rats in a time- and context-dependent manner [71]. The time intervals between substance administration and behavioral assessments also influence the outcomes of psychedelic substance studies. Although certain evidence suggests that acute DMT administration augments active coping strategies in the FST [74], psychedelics do not consistently exhibit rapid antidepressant effects [71], and behavioral changes indicative of antidepressant outcomes may only become measurable after four or more weeks following the psychedelic experience. Analogous to human responses, the role of “set and setting” seems to play a significant role in the sustained behavioral outcomes of rodents exposed to psychedelic substances. WKY rats administered a single dose of psilocybin (1 mg/kg) and subsequently assessed in the FST at various intervals between 1 and 5 weeks after treatment, as well as in the elevated plus maze 6 weeks post-administration, displayed distinct behavioral responses contingent on their initial exposure to the FST. Rats tested only once in the FST (one swim, 5 weeks after psilocybin) were notably more inclined to employ active coping strategies (swimming/climbing) than passive strategies (immobility) in that assay, yet they did not differ from control rats in their subsequent elevated plus maze behavior a week later. Conversely, rats subjected to weekly FST for five weeks (total of five swims) or tested at 1 and 5 weeks (two swims) exhibited only slight (yet statistically significant) increases in FST activity compared to control animals, while also displaying significantly reduced anxiety-like behavior in the elevated plus maze 6 weeks after psilocybin administration [71].

Regarding the outcomes of the antidepressant effects of ketamine observed in animal models of depression, the situation appears to be consistent. Ketamine and its metabolite (2R,6R)-HNK exhibit rapid antidepressant properties in animal models of TRD. In studies using the CMS model, the administration of a single dose of ketamine (10 mg/kg) to WKY rats at weekly intervals has been demonstrated to induce rapid antidepressant responses [38, 54]. Besides, the antidepressant efficacy of ketamine, even at lower doses (1, 3, and 5 mg/kg), as assessed through the FST, was discernible after an acute ketamine administration in WKY rats and this effect lasts for 24 h [75, 76].

Although ketamine produces rapid antidepressant effects, long-term antidepressant effects are produced by a single administration of psilocybin [71].

## What is known about the mechanisms of action of agents used in TRD therapy?

It appears that newly discovered therapeutic targets ultimately revolve around altering brain neuroplasticity. Therefore, the pursuit of novel therapeutic targets should be focused on those mechanisms whose regulation influences changes in brain neuroplasticity. Neural plasticity, also known as neuroplasticity or brain plasticity denotes the brain's capacity to adjust in reaction to regular developmental mechanisms, encounters, or injuries. This encompasses alterations in brain architecture like the generation of fresh neurons, the establishment of novel networks, and modifications within existing networks, specifically adjustments in synaptic potencies, culminating in shifts in functionality and conduct [77].

One of the main mechanisms that explain ketamine’s rapid antidepressant effects beyond simple NMDA antagonism is its ability to induce transient glutamate release and stimulate AMPA receptors, which may lead to the activation of processes associated with synaptic plasticity. As a result of this cascade, there can be an upregulation of the BDNF and activation of the tyrosine receptor kinase B (Trkβ receptor). The activated Trkβ receptor can influence the mTORC1 (target of rapamycin complex 1) signaling pathway, ultimately stimulating local protein synthesis and contributing to synaptic plasticity processes. A consequence of this activity could be the rapid proliferation of dendritic spines. Furthermore, it has been demonstrated that ketamine directly binds to Trkβ and allosterically enhances BDNF signaling [78].

The data indicating a direct interaction between glycogen synthase kinase 3 (GSK-3) and AMPA receptors in the antidepressant effects of ketamine are also intriguing [79, 80]. This association has been confirmed in previous studies on ketamine. It has been demonstrated that ketamine activates AMPA receptor signaling by inhibiting GSK-3 through a reduction in PSD-95 phosphorylation in the hippocampus [81]. In studies using GSK-3 knock-in mice, serine phosphorylation did not inhibit GSK-3; furthermore, the lack of GSK-3 inhibition was also associated with inducing depressive behaviors in response to various stressors [82]. Additionally, human studies have shown that ketamine deactivates GSK-3 activity (by lowering its phosphorylation) after a single bolus ketamine infusion in depressed patients [83]. Moreover, lithium—a potent GSK-3 inhibitor—enhanced the antidepressant effects of ketamine in mice. Lithium can also indirectly inhibit GSK-3 by activating the Akt kinase or by disrupting the β-arrestin complex [84, 85].

Recent studies indicate that in the WKY strain, mRNA of the gene encoding Gsk-3β is overexpressed in the prefrontal cortex, which could confirm the role of this protein in TRD [86]. Moreover, high GSK-3β activity is required for pre- and postsynaptic molecular mechanisms to support the occurrence of long-term depression (LTD) [87], which can be important for TRD. It seems, therefore, that the observed occurrence of the LTD phenomenon and the increased expression of Gsk3 β in WKY may have a significant impact on the response to antidepressant treatment in this model.

Generally, evidence data are indicating that WKY rats exhibit abnormalities in synaptic plasticity processes within key neural circuits relevant to depression. This strain shows reduced total hippocampal volume and impaired long-term potentiation (LTP) in the hippocampus, which may reflect an impairment of synaptic plasticity and function. Furthermore, a disrupted normal balance in hippocampal synaptic plasticity is observed in WKY rats [79]. Since LTP, which facilitates spine formation/enlargement, is significantly impaired in these animals, while long-term depression (LTD), associated with spine shrinkage/retraction, appears unchanged in the hippocampus, there might be a greater propensity for synaptic destabilization, loss of connectivity, and eventual neuronal atrophy in this crucial neural circuit implicated in MDD, potentially mediating or at least contributing to the structural and functional outcomes in the WKY strain [76]. It has been shown, that a single low dose of ketamine (5 mg/kg, i.p.) or its metabolite, (2R,6R)-HNK, reinstated LTP deficits in WKY rats after 3.5 h, but not after 30 min post-administration, with enduring effects still present at 24 h. This implies a delayed yet sustained facilitatory effect on synaptic plasticity in the hippocampus. Correspondingly, WKY rats exhibited compromised long-term spatial memory dependent on the hippocampus. Notably, both ketamine and (2R,6R)-HNK pre-treatment effectively restored this impairment. Conversely, in WKY rats that demonstrate maladaptive stress responses, ketamine, but not (2R,6R)-HNK, elicited rapid and persistent effects in the FST, a widely utilized preclinical measure of antidepressant-like activity [76].

Psychedelics appear to enhance neuroplasticity through the 5-HT2AR, which also mediates most of their subjective effects [88]. Although relatively low doses of the selective 5-HT2AR antagonist ketanserin do not fully block psychedelic-induced neuroplasticity [89], higher doses of ketanserin completely inhibit it [90]. Furthermore, the affinity of different psychedelic compounds for the 5-HT2AR predicts their individual potency as psychoplastogens (a class of compounds, that robustly promote structural and functional neural plasticity) [91], and mice lacking the 5-HT2AR show no signs of increased neuroplasticity following psychedelic treatment [90, 91]. Psychedelics stimulate postsynaptic 5-HT2AR on layer 5 and 6 pyramidal neurons as well as on GABAergic interneurons [88, 92]. The excitation of pyramidal neurons and increased extracellular glutamate levels lead to greater stimulation of AMPA receptors [92, 93]. The precise molecular pathways that may modify neuroplasticity after 5-HT2AR stimulation are not fully understood. Nevertheless, one hypothesis suggests that the aforementioned AMPA receptor stimulation initiates a positive feedback loop: AMPA receptor stimulation may enhance BDNF secretion, stimulating Trkβ receptors and mTOR, which, in turn, stimulate further BDNF production and sustained AMPA activation [90, 93, 94]. Sustained activation of both AMPA receptors and mTOR seems necessary for increased dendritic growth following psychedelic stimulation [90, 95]. Additionally, activity involving both 5-HT2AR and glutamate receptors, especially mGlu2, may be significant for psychedelics' effects on neuroplasticity [92, 96, 97].

On the other hand, recent studies indicate that psilocin—an active metabolite of psilocybin—demonstrates direct binding affinity to Trkβ. Interestingly, this affinity surpasses the binding affinity of conventional antidepressant drugs like fluoxetine and ketamine. Furthermore, the study reveals that the binding sites of psychedelics and antidepressants on Trkβ are distinct, albeit partially overlapping within the receptor’s transmembrane domain [98]. This study sheds light on the relationship between psychedelics and neurotrophic signaling, highlighting their impact on neuroplasticity and antidepressant-like behavior in murine models. It's noteworthy that these effects appear to be reliant on the interaction with Trkβ and the subsequent facilitation of endogenous BDNF signaling. Intriguingly, the activation of the 5-HT2AR does not seem to be the primary mediator of these effects, differentiating the mechanisms of psychedelics from those of other compounds.

Among other therapies, repetitive transcranial magnetic stimulation (rTMS), which is an effective therapy for TRD [99], also seems to influence brain neuroplasticity and specifically induces changes in the hippocampal [100] and in the cortical excitability [101]. In the study involving 31 patients with chronic TRD who received either active high-frequency rTMS or sham treatment over four consecutive weeks, significant changes in cortical thickness between TRD treatment and sham patients occurred. Moreover, longitudinal changes in amygdala volume were identified in males [102].

Studies on rats using VNS method in TRD therapy demonstrated that acute VNS led to the upregulation of neurotransmitters, including norepinephrine, and increased expression of neurotrophic factors, which play role in neuroplasticity, such as BDNF and fibroblast growth factor (bFGF), in the hippocampus and cerebral cortex [103]. Additionally, a study on TRD patients found that VNS was associated with increased hippocampal volumes, mirroring clinical improvement, suggesting the importance of hippocampal volumes as a marker for VNS response in TRD [56, 104].

**Fig. 1:**
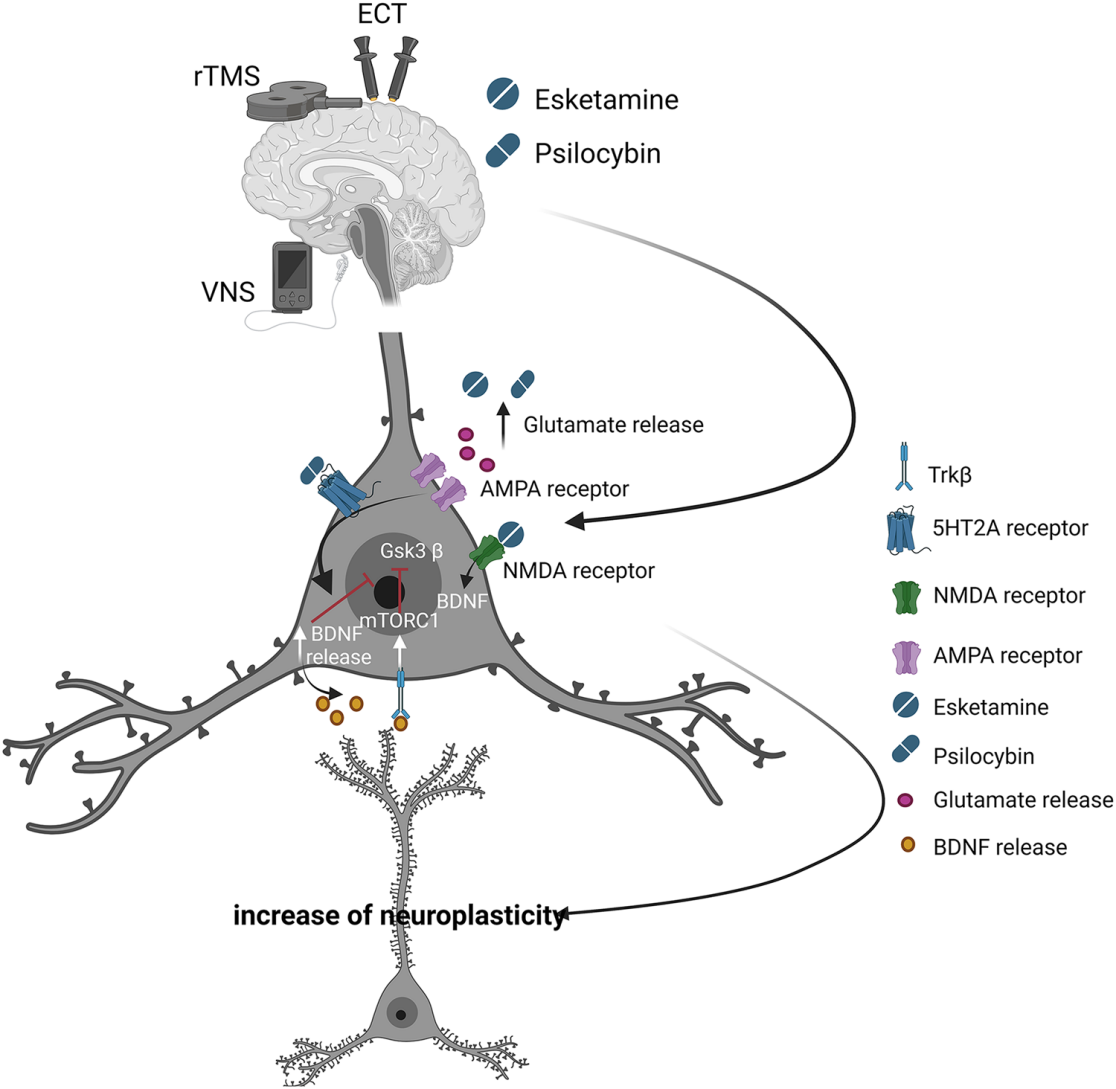
Approved therapies for treatment-resistant depression and their potential mechanisms of action based on [80, 110, 111]. Created with BioRender.com

Structural imaging studies in patients undergoing ECT primarily focus on subcortical regions, such as the hippocampus and amygdala, due to their potential associations with adult neurogenesis occurring in the dentate gyrus of the hippocampus [105, 106]. Several groups have demonstrated that ECT leads to increased volumes of the hippocampus and amygdala [107, 108] and observed a correlation with symptom improvement [109]. Furthermore, macrostructural changes at the cortical level have also been documented and the results confirm that neuroplasticity occurs within the network of cortical areas to promote favorable therapeutic outcomes [110] (Fig. 1).

## New perspectives on TRD therapy

Recent investigations on WKY rats have unveiled the potential mitigation of CMS-induced impairments through interventions like deep brain stimulation (DBS), consequently imparting new perspectives on TRD treatment [38, 54, 112, 113]. Furthermore, this model demonstrated that Wistar rats unresponsive to repeated administrations of citalopram or venlafaxine exhibit reactivity to these antidepressant drugs following the application of deep ventromedial prefrontal cortex stimulation [114]. These findings substantiate the concept that DBS targeting the prefrontal cortex can yield efficacy within a rat model exhibiting resistance to chronic antidepressant treatment. This replication mirrors the clinical outcome of DBS in cases of TRD.

It was also shown in animal models that the antidepressant effect induced by DBS on lateral habenula nuclei (LHb) was associated with changes in Ca^2+^/calmodulin-dependent protein kinase (CaMKIIα/β), GSK3α/β and AMP-activated kinase (AMPK) [115]. The study on WKY rats revealed that certain miRNAs (miR-133a, miR-708, and miR-92a) exhibited significant differentiation between WKY and WIS rats [116] and increased expression of miR-203a and miR-708 in WKY rats corresponded with decreased levels of Slc12a5 mRNA expression. The Slc12a5 gene encodes the neuronal KCC2 channel (potassium-chloride transporter member 5), a key regulator of intracellular chloride levels in mature neurons, and also regulates spine density [117]. Altered KCC2 function has implications for neuronal inhibition, as reduced expression has been linked to various neurological and psychiatric conditions, often leading to depolarized GABAAR-mediated currents [118]. Interestingly, studies indicate that in patients with TRD, stimulation of the LHb with DBS showed therapeutic effects that correlated with periods of active stimulation [119].

Furthermore, recent research indicates that preferential activation of cortical 5-HT1A receptors, through the use of biased agonists such as NLX-101 or NLX-204, also exerts antidepressant effects in WKY rats, thus potentially representing an innovative treatment strategy for TRD [38].

## Conclusion

Treatment-resistant depression is challenging to define, and the confluence of multiple factors can further complicate the accurate diagnosis of this condition. Consequently, identifying specific biomarkers responsible for treatment resistance also remains elusive. Nevertheless, animal models of TRD, such as the chronic mild stress (CMS) model and the WKY strain, appear to serve as reliable and well-validated models of TRD, offering a potential avenue to uncover specific targets and novel therapeutic approaches for this disorder.

The exploration of new therapeutic targets revolves around manipulating brain neuroplasticity. This pursuit is underlined by the understanding that neural plasticity enables the brain to adapt to developmental processes, experiences, and injuries, involving changes in neural architecture and synaptic strengths. Mechanisms such as ketamine’s impact on AMPA receptors, its influence on BDNF and Trkβ signaling pathways, as well as the enhancing effect of psychedelics on neuroplasticity through the 5-HT2AR, all contribute to synaptic changes associated with depression and antidepressant effects. Additionally, the interaction between GSK-3 and AMPA receptors in ketamine’s antidepressant action, and the ability of psilocin to bind directly to Trkβ, highlight potential targets for intervention. Repetitive rTMS or ECT, an effective therapy TRD, also prompts investigations into its effects on brain neuroplasticity. In the context of the search for new therapies related to neuroplasticity, the results of the previously discussed GWAS studies appear to be particularly intriguing. These insights collectively offer promising avenues for the development of novel therapeutic strategies for TRD.

## Data Availability

Not applicable.

## Abbreviations

5-HT1AR: Serotonin receptor type 1
5-HT2AR: Serotonin receptor type 2
5-HTTLPR: Serotonin-transporter-linked polymorphic region
ABCB1: ATP-binding cassette subfamily B member 1
AMPK: AMP-activated kinase
BDNF: Brain-derived neurotrophic factor
C3: Complement component 3
CaMKIIα/β: Ca^2^^+^/calmodulin-dependent protein kinase
CANMAT: Canadian Network for Mood and Anxiety Treatments
CMS: Chronic mild stress
COMT: Catechol-*O*-methyltransferase
CR1L: Complement C1q receptor
CUMS; UCMS: Chronic unpredictable mild stress
CVS: Chronic variable stress
CYP2C19: Cytochrome P450 2C19
CYP2D6: Cytochrome P450 2D6
CYP450: Cytochrome P450
DBS: Deep brain stimulation
DMT: *N*,*N*-Dimethyltryptamine
ECT: Electroconvulsive therapy
EIF2AK2: Eukaryotic translation initiation factor 2 alpha kinase 2
ESM: European Staging Method
FDA: U.S. Food and Drug Administration
FST: Forced swim test
GIRK4: Glutamate ionotropic receptor kainate type subunit 4
GRIN2B: Glutamate ionotropic receptor NMDA type subunit 2B
GSK-3: Glycogen synthase kinase 3
GSK-3β: Glycogen synthase kinase-3 beta
GWAS: Genome-wide association study
KCC2: Potassium chloride transporter member 5
LH: Learned helplessness
LHB: Lateral habenula nuclei
LSD: Lysergic acid diethylamide
LTB4R: Leukotriene B4 receptor
LTD: Long-term depression
LTP: Long-term potentiation
MAOIs: Monoamine oxidase inhibitors
MDD: Major depressive disorder
MGH: Massachusetts General Hospital Staging Method
mTORC1: The target of rapamycin complex 1
NMDA: *N*-Methyl-d-aspartate receptor
OBX: Olfactory bulbectomy model
P-gp: P-glycoprotein
PPP3CC: Protein phosphatase 3 catalytic subunit gamma
PRKRA: Protein activator of interferon-induced protein kinase
PYHIN1: Pyrin and HIN domain family member 1
rTMS: Repeated transcranial magnetic stimulation
SHR: Spontaneously hypertensive rat
Slc12a5: Solute carrier family 12 member 5
SLC6A2: Solute carrier family 6 member 2
SLC6A4: Solute carrier family 6 member 4
SLC7A8: Solute carrier family 7 member 8
SSRIs: Selective serotonin reuptake inhibitors
STARD: Sequenced Treatment Alternatives to Relieve Depression
STK19: Serine/threonine kinase 19
SV2A: Synaptic vesicle glycoprotein 2A
TCAs: Tricyclic antidepressants
TGA: Therapeutic Goods Administration
TRD: Treatment-resistant depression
Trkβ: Tyrosine receptor kinase B
TST: Tail suspension test
VNS: Vagus nerve stimulation
WHO: World Health Organization
WKY: Wistar Kyoto rat
ZDHHC3: Zinc finger DHHC-type palmitoyltransferase 3
ZNF248: Zinc finger protein 248
